# The Epileptic Colony at Chalfont St. Peter

**Published:** 1895-12-07

**Authors:** 


### THE EPILEPTIC COLONY AT CHALFONT ST. PETER.

### OPENING OF A NEW HOME.

The Colony for Epileptics at Chalfont St. Peter, Bucks, established not much more than a year ago on the lines of that at Bielefeld, is steadily extending its most important and useful work. The site, a farm of 135 acres, was originally provided through the generosity of Mr. Passmore Edwards, and on this land temporary buildings were erected for the accommodation of 18 men. Last November the foundation-stone was laid of a permanent building, which was opened by the Duke of Devonshire on Tuesday, November 26th. The entire expense of this home has been also defrayed by Mr. Passmore Edwards.

A good many visitors journeyed down to Chorley Wood (the nearest station) on Tuesday to take part in the proceedings, and the long procession of vehicles of many and various descriptions which conveyed them over the four miles of country lanes to the colony must have proved quite an excitement to the ordinary inhabitants. Sir William and Lady Broadbent were there, Mr. and Mrs. Passmore Edwards, General Moberly, Dr. Ferrier, Dr. Savage, and Dr. Buzzard, and many others. A tent was put up in the grounds, and here, after a short dedicatory service, the Duke of Devonshire declared the new building to be formally open. As a matter of fact, it has been occupied since August, though circumstances had prevented the formal notification of the fact until now. Mr. Montefiore Nicholls, the chairman of the Executive Committee, in his opening address, was able to make the glad announcement that Mr. Passmore Edwards had promised to continue his interest in the colony by the erection of two homes for children of both sexes, and that another generous donor had undertaken to provide one for women, this latter to be begun as early in the coming year as the weather might permit.

The Duke of Devonshire, in declaring the new building open, said he felt that it was especially important that public attention should be called to the work of the Society for the Employment of Epileptics, having regard to the large number of epileptics in this country, some 30,000 or 40,000, and the piteousness of their unaided condition. He commented upon the particular advantages to be derived in the case of epileptics from regular and healthful employment, and pleasant social intercourse in their leisure hours. The home now opened was of an unpretentious character, but the importance of the work must not be judged by the size of the present establishment; there could be no reason why such colonies should not be established in other parts of the country. Cordial recognition must be tendered to Mr. Passmore Edwards for his munificent help. It was not the first occasion by very many that he had devoted part of his fortune to the alleviation of the sufferings of his fellow-men, and it was to be hoped that his splendid example might be followed by others.

Sir William Broadbent proposed a vote of thanks to the Duke and Duchess of Devonshire for their presence, after which the visitors adjourned to the home for tea.

The new building provides accommodation for 18 men, raising the total number now employed in the colony to 36. There are two large dormitories, with nine beds in each, with a room for a male attendant between, communicating with both. The matron's and nurses' bed-rooms, bath-room, &c., are shut off entirely from this part of the building. Downstairs there is a good-sized dining-room, with a smaller recreation-room—prettily decorated with painted panels by the Kyrle Society—opening from it, kitchen and offices, and the matron's sitting-room and a small sitting-room for general purposes. The baths, lavatories, and sanitary fittings generally are adequate and complete.

The result, from a medical point of view, of the establishment of this scheme has been most satisfactory. The inmates of the colony invariably improve in health, and their fits decrease in number and severity. Association with others suffering from the same malady has been proved to have the best effect upon epileptics. In ordinary life they are tempted to brood over their own condition, but "at the colony they see their neighbours suffering under the same affliction, the disease is no longer peculiar to themselves, and ceases to be the important factor in their lives." The colonists live happily together; good progress is being made with the cultivation of the farm and garden land, the latter being entirely tilled by the colonists themselves, and supplying plenty of fresh vegetables. The future work of the society has apparently no limit, and no scheme can more appropriately call itself "National," for the needs of epileptics have cried out for alleviation for long, and the results to be achieved are great. It must be remembered that funds are much needed for the adequate carrying out of the work, for each extension of the scheme entails considerable expenditure. At present there is no resident medical officer, but such an appointment will sooner or later become imperative, and suitable accommodation will have to be provided, and a laundry for the employment of the women will be an early necessity. Gifts in kind are earnestly asked for, games, books, and especially clothing, old or new, and a waggonette is much needed to give the colonists the pleasure of an occasional drive through the lovely country lanes. Visitors to the colony are welcomed at any time, and a personal inspection could not fail to arouse interest and sympathy. The route is from Baker Street to Chorley Wood, where carriages can always be obtained for the pretty drive to Chalfont St. Peter.

